# Curcumin’s Protective Effects Against H_2_O_2_- and AAPH-Induced Oxidative Damage in Red Blood Cells: Mechanisms, Evidence Synthesis, and Perspectives on Translational Applications

**DOI:** 10.3390/molecules31142464

**Published:** 2026-07-14

**Authors:** Tianzhu Yu, Fengyan Hou, Xiyao Yin, Jianjun Dong, Xia Wang, Jie Jiao, Zuobin Wang

**Affiliations:** 1 International Research Centre for Nano Handling and Manufacturing of China, Changchun University of Science and Technology, Changchun 130022, China; 15044225079@163.com (T.Y.); yinxiyao0330@163.com (X.Y.); jj805748434@163.com (J.J.); 2 Centre for Opto/Bio-Nano Measurement and Manufacturing, Zhongshan Institute of Changchun University of Science and Technology, Zhongshan 528437, China; 3 Ministry of Education Key Laboratory for Cross-Scale Micro and Nano Manufacturing, Changchun University of Science and Technology, Changchun 130022, China; 4 Department of Physics and Electronic Engineering, Jinzhong University, Jinzhong 030600, China; 18635489198@163.com; 5 School of Electronic Information and Artificial Intelligence, West Anhui University, Lu’an 237012, China; 03260168@wxc.edu.cn; 6 School of Electronic Information Engineering, Henan Institute of Technology, Xinxiang 453000, China; 2021200032@mails.cust.edu.cn; 7 Joint Research Centre for Computer-Controlled Nanomanufacturing, University of Bedfordshire, Luton LU1 3JU, UK

**Keywords:** curcumin, red blood cells, oxidative stress, H_2_O_2_, AAPH, hemolysis, eryptosis, band 3, blood storage

## Abstract

Red blood cells (RBCs) are the most abundant cells in peripheral blood and perform critical functions including oxygen and carbon dioxide transport, acid base buffering, regulation of hemorheology, and modulation of immune signaling. Due to their high content of hemoglobin and labile iron, prolonged exposure to high oxygen tension, membrane enrichment with polyunsaturated fatty acids, and the absence of both nucleus and mitochondria, mature RBCs have limited capacity for damage repair and protein re-synthesis, making them highly susceptible to attack by reactive oxygen species (ROS) and reactive nitrogen species (RNS). Hydrogen peroxide (H_2_O_2_) and 2,2′-azobis(2-methylpropionamidine) dihydrochloride (AAPH) are the two most commonly used inducers in the in vitro models of RBC oxidative injury: H_2_O_2_ primarily generates hydroxyl radicals via hemoglobin/ferrous ion-dependent Fenton reactions, simulating acute oxidative stress. AAPH releases peroxyl radicals upon thermal decomposition, mimicking persistent lipid peroxidation in cell membranes. Curcumin, a representative polyphenolic compound derived from turmeric, exerts multiple effects including free radical scavenging, metal ion chelation, membrane stabilization, anti-inflammatory activity, and regulation of redox homeostasis. This review systematically summarizes the pathological basis of RBC oxidative damage and the protective effects of curcumin on membrane systems, antioxidant defenses, morphology, and function, based on the core evidence chain “H_2_O_2_/AAPH—RBCs—curcumin”, integrating recent experimental findings on H_2_O_2_, AAPH, blood storage-induced injury, and curcumin formulations. It emphasizes that mature RBCs lack nuclei and mitochondria, and therefore mechanisms such as Nrf2/ARE signaling, HO-1 induction, mitochondrial apoptosis, caspase cascades, and inflammasome activation should not be directly equated with transcriptional regulatory pathways within mature RBCs, but rather interpreted as indirect evidence originating from nucleated cells, erythroid progenitors, or the blood microenvironment. The article further proposes that future research should focus on standardized RBC models, physiologically relevant dosages, nanodelivery systems, and translational applications in blood storage, to facilitate the transition of curcumin’s in vitro antioxidant evidence into clinical transfusion medicine and precision nutritional interventions.

## 1. Introduction

Red blood cells are the most abundant cells in peripheral blood and are continuously exposed to high oxygen tension. Their oxygen-carrying function, membrane lipid composition, and iron-dependent reaction jointly determine their high sensitivity to oxidative stress [1]. Mature red blood cells lack a nucleus and mitochondria and cannot rapidly repair damage through transcriptional regulation and protein re-synthesis. Therefore, they mainly rely on existing antioxidant systems such as glutathione, catalase, superoxide dismutase, glutathione peroxidase, and peroxiredoxin to maintain homeostasis [2]. When reactive oxygen species production exceeds the defense threshold, red blood cells can undergo changes such as hemoglobin oxidation, membrane lipid peroxidation, band 3 and membrane skeleton protein modification, decreased deformability, phosphatidylserine flipping, and hemolysis [3,4,5,6,7]. These changes not only affect the lifespan and oxygen delivery efficiency of red blood cells but are also closely related to pathological processes such as inflammation, microcirculation disorders, coagulation activation, and blood storage damage [8,9,10].

To model RBC oxidative injury in vitro, H_2_O_2_ and AAPH are commonly used as complementary inducers. H_2_O_2_ can enter red blood cells and react with hemoglobin, heme iron or Fe^2+^, forming hydroxyl radicals and high-valent hemoglobin oxidation products, suitable for simulating acute, iron-dependent oxidative attacks [1]. AAPH thermally decomposes at 37 °C to generate peroxyl radicals, making it more suitable for modeling membrane lipid peroxidation and delayed hemolysis. Curcumin, as a representative polyphenolic compound derived from turmeric, possesses characteristics such as phenolic hydroxyl group donation, β-diketone structure metal chelation, membrane localization, and lipid peroxidation chain reaction inhibition. Theoretically, it can simultaneously intervene in H_2_O_2_-related hemoglobin/iron-dependent damage and AAPH-related peroxide radical membrane damage [11,12]. Studies have shown that curcumin can reduce the red blood cell hemolysis and lipid peroxidation induced by H_2_O_2_ or AAPH, maintain antioxidant indicators such as GSH and SOD, and affect key nodes such as ferrylHb, ATP, and band 3 phosphorylation under blood storage or peroxidation damage conditions [13,14,15,16].

Although curcumin’s antioxidant effects have been widely reported, its mechanisms in mature RBCs require careful evidence stratification. Mature red blood cells do not have a nucleus or mitochondria, so mechanisms such as Nrf2/ARE transcriptional activation, HO-1 expression upregulation, mitochondrial membrane potential changes, and the classical caspase cascade cannot be indiscriminately regarded as direct mechanisms within mature red blood cells, but should mainly be considered as indirect support in erythroid precursor cells, nucleated blood cells, endothelial cells, or the systemic blood microenvironment [7,11,12]. This review therefore focuses on the evidence chain “H_2_O_2_/AAPH-induced oxidative damage–RBC structural and functional changes–curcumin protection” and distinguishes direct RBC evidence from indirect evidence obtained from nucleated cells or systemic models [13,14,15,16,17,18,19,20].

This article is a narrative evidence integration review rather than a quantitative Meta-analysis. The literature was mainly retrieved from PubMed, Web of Science, Scopus, Google Scholar, and CNKI. The search period ended in May 2026. The search terms included “curcumin”, “erythrocyte” or “red blood cell”, “H_2_O_2_”, “hydrogen peroxide”, “AAPH”, “oxidative stress”, “hemolysis”, “eryptosis”, “band 3”, “ferryl hemoglobin”, “blood storage”, and “nanodelivery”, etc. Studies included needed to involve red blood cell oxidative damage, curcumin or curcumin preparations, H_2_O_2_/AAPH-related models, blood storage damage or mechanisms related to antioxidant pathways. Exclusion criteria included: models without red blood cell-related endpoints, no evaluation of curcumin, or mechanisms discussed only in the nucleated cell system unrelated to red blood cell oxidative damage. The evidence was classified as: (1) isolated human/mammalian mature red blood cells with direct in vitro or in vivo evidence; (2) direct evidence of stored human red blood cells; (3) direct cellular evidence of avian nucleated red blood cells, but not equivalent to mammalian mature red blood cell mechanism evidence; (4) indirect in vivo evidence from human or animal supplementation studies; (5) indirect mechanism evidence from other nucleated cells or erythroid precursor cells; (6) no evidence of cell chemistry and preparation/carrying support.

## 2. Oxidative Stress and Red Blood Cell Damage

### 2.1. Pathophysiological Basis of Oxidative Stress

Oxidative stress refers to the imbalance between the generation of oxidants and the antioxidant defense in the body, resulting in abnormal accumulation of ROS/RNS in cells and tissues, and triggering the oxidative modifications of biological macromolecules such as lipids, proteins, carbohydrates, and nucleic acids [21]. Obeagu summarized the systemic effects of oxidative stress on red blood cells from aspects such as lipid peroxidation of the red blood cell membrane, oxidation of hemoglobin, depletion of antioxidant enzymes, and the risk of hemolysis, and pointed out that red blood cells are both the target cells of oxidative damage and an important window for observing the oxidative–reductive state of the body [22]. Spinelli et al. in their review on the red blood cell redox homeostasis further emphasized that the high hemoglobin content and high oxygen exposure of red blood cells naturally place them in an oxidative challenge, and glutathione, catalase, superoxide dismutase, peroxiredoxin 2, and glutathione peroxidase together constitute its main defense line [23]. From a pathophysiological perspective, oxidative stress is not merely a “damage event”, but a dynamic network coupled with inflammation, metabolic reprogramming, endothelial dysfunction, coagulation activation, and cell death. Orrico et al. pointed out that during oxidative stress in healthy and diseased states, hemoglobin auto-oxidation, membrane-bound hemoglobin, free heme, iron ions, and peroxides could form a self-amplifying oxidative cycle [24]. Spinelli et al. starting from the red blood cell metabolic pathways summarized that oxidative stress affected glycolysis, the pentose phosphate pathway, the Rapoport–Luebering bypass, the glutathione cycle, and purine metabolism, thereby converting “decreased antioxidant capacity” into “decreased energy metabolism and membrane stability” [25]. These studies suggest that the red blood cell oxidative damage is the result of changes in the red blood cell membrane, hemoglobin, metabolic enzymes, and ion transport systems.

### 2.2. Susceptibility of Red Blood Cells to Oxidative Damage

The susceptibility of red blood cells to oxidative damage comes from three levels. First, red blood cells undertake the function of oxygen transport, and hemoglobin can undergo auto-oxidation and generate superoxide anions and H_2_O_2_ during the oxygenation and deoxygenation cycle. Second, the red blood cell membrane is rich in polyunsaturated fatty acids, which are easily attacked by hydroxyl radicals and peroxide radicals, forming lipid radicals, lipid peroxides, and MDA. Third, mature red blood cells have no nucleus or mitochondria and cannot rapidly replenish damaged proteins through classical transcriptional regulation, so they relied more on existing enzyme systems and low-molecular antioxidants [1,24,26]. Remigante et al. summarized with band 3 as the core that band 3 was not only responsible for Cl^−^/HCO_3_^−^ exchange but also connected the membrane skeleton and regulated the localization of hemoglobin and glycolytic enzymes, while its oxidative modification affected morphology, deformability, and metabolic homeostasis [27].

Daraghmeh et al. summarized the red blood cell redox process as a closed loop: when systems such as SOD, CAT, GSH-Px, and PRDX2 promptly cleared ROS, red blood cells maintained a biconcave disk shape and high deformability. When the oxidative load exceeded the defense threshold, hemoglobin was oxidized to ferryl hemoglobin or ferrylHb, membrane proteins underwent carbonylation, sulfhydryl oxidation, cross-linking, or tyrosine phosphorylation, and ultimately manifested as increased osmotic fragility, decreased deformability, PS externalization, and hemolysis [28]. Möller et al. from the perspective of red blood cell redox biochemistry pointed out that the fate of H_2_O_2_ in red blood cells depended on CAT, GPx, PRDX2, and the competition reaction with hemoglobin, and thus the same oxidant produced protective signals or irreversible damage under different doses and times [29].

### 2.3. Advantages of Red Blood Cells as an Oxidative Damage Model

Red blood cells are an important in vitro model for evaluating natural antioxidants. The classic study by Mohanty et al. emphasize that oxidative stress in red blood cells can directly reduce membrane deformability and affect oxygen delivery, and this process is related to cellular senescence, macrophage clearance, and microcirculation perfusion efficiency [30]. Compared with cultured nucleated cells, the red blood cell model has the advantages of easy availability of sources, relatively simple structure, clear experimental endpoints, and lower ethical burden. Common endpoints include hemolysis rate, MDA/TBARS, ferrylHb, GSH/GSSG, SOD/CAT/GSH-Px activity, membrane protein carbonylation, band 3 function, cell morphology, and PS externalization [31].

It is worth noting that the “simplicity” of the red blood cell model does not imply a single mechanism. Tkachenko and Havránek point out in their discussion on eryptosis that mature red blood cells lack mitochondria and nuclei, but still can undergo a regulatory death-like process characterized by Ca^2+^ signaling, cell shrinkage, vesicle formation, and PS externalization. They also suggest being cautious in the use of terms to distinguish eryptosis, hemolysis, necrotic-like lysis, and nucleated cell apoptosis [32]. Therefore, red blood cells can be an ideal model for studying membrane protection of antioxidants and eryptosis, but Nrf2 nuclear translocation, mitochondrial membrane potential, and transcriptional regulation mechanisms in nucleated cells should not be directly applied to mature red blood cells without qualification.

**Figure 1:**
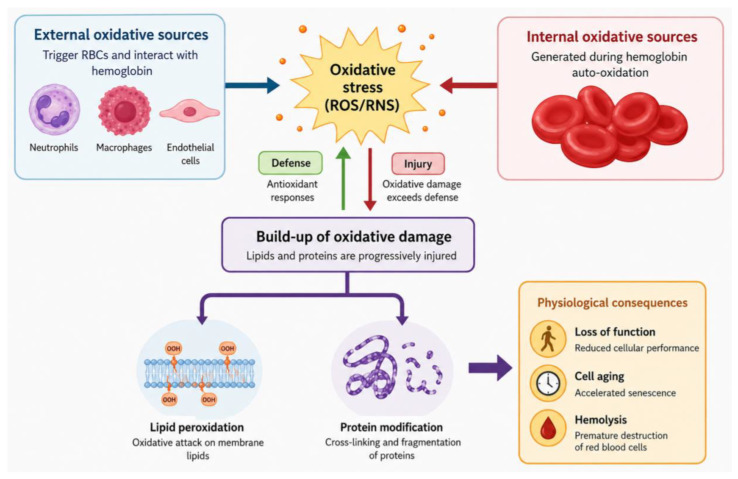
Overview of ROS/RNS generation and oxidative damage in red blood cells. Exogenous oxidants from neutrophils, macrophages, and endothelial cells, together with endogenous oxidants from hemoglobin auto-oxidation, can overwhelm RBC antioxidant defenses. The resulting lipid peroxidation and protein modification impair RBC function, accelerate senescence, and may lead to hemolysis.

The polyunsaturated fatty acid-rich red blood cell membrane is highly susceptible to oxidative damage, reducing oxygen-carrying capacity and aggravating tissue hypoxia [33]. ROS oxidize hemoglobin, induce lipid peroxidation, and damage membrane and cytoskeletal proteins, thereby impairing red blood cell morphology, deformability, and lifespan while increasing aggregation and blood viscosity [34]. These changes contribute to cardiovascular and cerebrovascular diseases. Figure 1 shows the effects of ROS on the body.

## 3. Oxidative Damage Inducers: H_2_O_2_ and AAPH

### 3.1. Mechanism and Characteristics of H_2_O_2_-Induced Oxidative Damage

**Figure 2:**
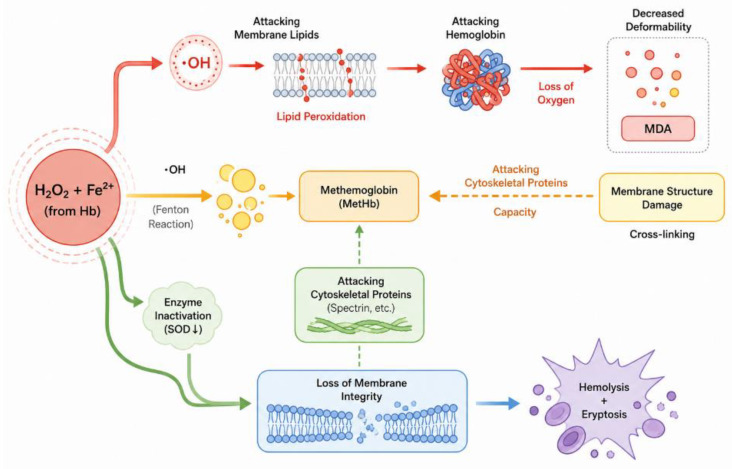
H_2_O_2_ induces oxidative erythrocyte injury through Fe^2+^-driven hydroxyl radical formation. These radicals promote membrane lipid peroxidation, hemoglobin oxidation to methemoglobin, protein damage, elevated MDA, and reduced deformability and oxygen-carrying capacity. Antioxidant enzyme inhibition further compromises membrane integrity, ultimately causing eryptosis and hemolysis.

H_2_O_2_ is one of the most commonly used inducers in the in vitro acute oxidative damage model of red blood cells. It has a small molecular size and can pass through the biological membrane. It reacts with hemoglobin, ferrous ions, and the peroxidase system within the red blood cells. In the presence of Fe^2+^ or heme iron, H_2_O_2_ can generate hydroxyl radicals through the Fenton reaction. These radicals are highly reactive and can rapidly attack membrane lipids, protein sulfhydryl groups, hemoglobin, and membrane structural components [35]. These radicals mainly damage the structure and function of red blood cells through three pathways (Figure 2). Remigante et al. evaluated the antioxidant activity of quercetin using a human red blood cell H_2_O_2_ model. The method involved detecting ROS, osmotic fragility, MDA, GSH, antioxidant enzymes, and morphological changes. The results confirmed that H_2_O_2_ induced oxidative damage in red blood cells within a short period of time, and natural polyphenols partially restored the homeostasis of red blood cells [36,37].

In the research related to curcumin, Benincasa et al. established an H_2_O_2_-induced model for accelerating red blood cell senescence, and compared the effects of curcumin, vitamin C, vitamin E, and combined treatments on the hemolysis rate and TBARS of red blood cells. The results showed that the pretreatment with 100 μM curcumin could significantly reduce H_2_O_2_-induced hemolysis and was superior to vitamin C or vitamin E alone at certain endpoints, suggesting that curcumin not only had free radical scavenging ability but might also exert a protective effect on the red blood cell membrane through membrane interaction [38]. The advantages of this H_2_O_2_ model are rapid reaction, clear endpoints, and good repeatability. The disadvantages are that high-dose H_2_O_2_ may not fully simulate the chronic oxidative environment in the body, and the concentrations of H_2_O_2_, treatment time, and red blood cell concentrations vary greatly in different experiments, making it difficult to directly compare the results.

### 3.2. The Mechanism and Characteristics of AAPH-Induced Oxidative Damage

AAPH is a water-soluble azo compound that can stably release free radicals upon thermal decomposition at 37 °C, and further react with oxygen to form peroxide free radicals. Unlike H_2_O_2_, AAPH does not rely on metal ion catalysis and is more inclined towards continuous membrane lipid peroxidation and peroxide free radical chain reactions. It is commonly used to evaluate the ability of antioxidants to block peroxide free radical-induced hemolysis [39]. Banerjee et al. studied the effect of curcumin on AAPH-induced hemolysis of human red blood cells, including AAPH-induced red blood cell oxidation, hemolysis determination, lipid peroxidation, K^+^ release, and GSH depletion. The conclusion was that curcumin exhibited a concentration-dependent antioxidant/pro-oxidant bidirectional effect. At low to medium concentrations, it could protect red blood cells, while higher concentrations might enhance oxidative damage [40].

Zhang et al. used AAPH to attack chicken red blood cell models and compared the free radical scavenging ability and red blood cell protection effect of seven natural pigments. They found that curcumin could reduce the hemolysis rate, decrease MDA, and increase T-SOD activity, and showed time and dose dependence [41]. Subsequently, Zhang et al. further compared the antioxidant spectra of curcumin and bisdemethoxycurcumin (BDMC). The methods included the in vitro red blood cell oxidative damage and detection of indicators in broiler chickens. The results suggested that curcumin was superior to BDMC in maintaining SOD activity, reducing MDA, and inhibiting hemolysis [42]. These results constitute the classic direct evidence of curcumin in the AAPH red blood cell model. These results should be divided into two levels of evidence: the in vitro experiment of chicken red blood cells belongs to “direct cellular evidence of bird nucleated red blood cells”, which can directly support the phenotypes of chicken red blood cells such as hemolysis, MDA and T-SOD. However, because chicken mature red blood cells retain nuclei and mitochondria, they cannot be used as direct evidence of Nrf2/HO-1, mitochondria or classical apoptosis mechanism in mature mammalian red blood cells. The whole experiment of broilers belongs to “indirect evidence in vivo”, and its effect may be affected by absorption, metabolism and tissue microenvironment at the same time. Therefore, both types of evidence should be interpreted separately from the direct evidence of human mature red blood cells and stored human red blood cells.

### 3.3. Comparison and Complementarity of the Two Induction Model Systems

**Figure 3:**
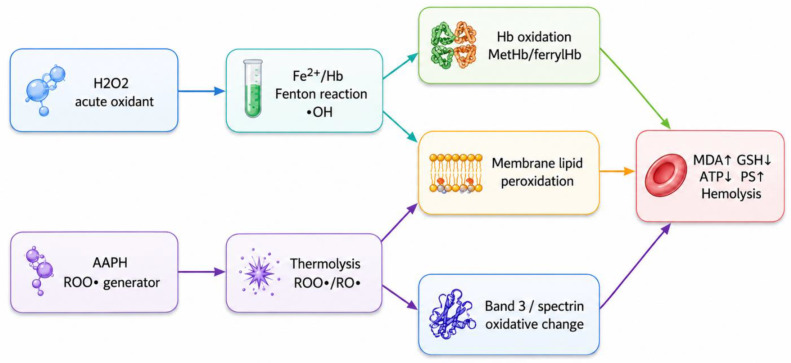
Complementary H_2_O_2_- and AAPH-induced oxidative injury pathways in RBCs. H_2_O_2_ mainly drives Fe^2+^/heme-dependent hydroxyl radical formation and hemoglobin oxidation, whereas AAPH continuously generates peroxyl radicals that promote membrane lipid peroxidation. Both routes converge on membrane protein damage, ATP/GSH depletion, PS externalization, and hemolysis.

The complementarity of the H_2_O_2_ and AAPH models lies in the sources of free radicals, damage kinetics, and main targets. The H_2_O_2_ model is more suitable for simulating short-term, acute, hemoglobin/iron-dependent oxidative damage, and is more advantageous in detecting ferrylHb, band 3 phosphorylation, membrane protein oxidation, and early hemolysis (Figure 3). Hicks et al. used a new curcumin gel formulation to treat peroxide damage and stored red blood cells, and found that this formulation could reduce pro-oxidative ferrylHb, restore ATP, and prevent tyrosine phosphorylation at the band 3 site, indicating that H_2_O_2_-related damage was used to capture the connection between hemoglobin oxidation, membrane protein modification, and energy metabolism decline [43]. The AAPH model was more suitable for simulating the continuous generation of peroxide free radicals and the process of membrane lipid peroxidation. Kang et al. studied the effect of PVA on the solubility, stability, and biological activity of curcumin, and detected the free radical scavenging ability of DPPH, ABTS, AAPH, and NO. The results showed that PVA significantly improved the water dispersibility, light stability, and AAPH free radical scavenging activity of curcumin [44]. Nuruki et al. from a methodological perspective pointed out that AAPH oxidizes oxyhemoglobin to ferrylHb, so using 540 nm absorbance in AAPH-induced hemolysis experiments might underestimate or misinterpret hemolysis, and should prefer more suitable detection wavelengths such as 523 nm. This study has important reference value for the subsequent standardization of the AAPH red blood cell model (Table 1) [45].

## 4. Research Overview of Curcumin

### 4.1. Chemical Structure and Physical Properties of Curcumin

Curcumin is the main bifunctional phenolic compound in the rhizome of turmeric. Its structure contains two adjacent methoxy phenolic hydroxyl groups and a β-diketone/epoxide interchange structure. The phenolic hydroxyl groups can accept hydrogen and stabilize the phenolic radical, and the β-diketone structure can form complexes with transition metal ions. Therefore, curcumin not only has chain-breaking antioxidant activity but also has certain potential for metal ion chelation. At the same time, curcumin is highly hydrophobic, has poor water solubility, is unstable under light and alkaline conditions, and has limited absorption and systemic exposure after oral administration. These problems restrict its in vivo transformation [46]. Pandey et al. studied the reduction metabolites of curcumin and pointed out that metabolites such as tetrahydrocurcumin and hexahydrocurcumin may play an important role in the antioxidant activity in the body [47]. Hewlings and Kalman also emphasized that the low bioavailability of curcumin was the core bottleneck of its clinical application [48].

### 4.2. Antioxidant and Membrane Protection Activity of Curcumin

The antioxidant activity of curcumin has multi-level characteristics: first, it directly eliminates free radicals such as DPPH, ABTS, peroxide free radicals, hydroxyl free radicals, and NO. Second, it chelates metal ions such as Fe^2+^, reducing the intensity of the Fenton reaction. Third, it embeds into the lipid bilayer through hydrophobic interactions, changing the lipid microenvironment and blocking the propagation of lipid peroxidation chain. Fourth, it can regulate signaling pathways such as Nrf2/ARE, NF-κB, MAPK, and NOX in nucleated cells [49]. Cui et al. summarized the antioxidant stress mechanism of curcumin, believing that it mainly maintained redox balance by eliminating ROS, enhancing the activity of antioxidant enzymes, inhibiting lipid peroxidation, and chelating metal ions, and could also regulate Keap1-Nrf2/ARE and NF-κB pathways in nucleated cells [50].

### 4.3. Hotspots of Curcumin in the Study of Red Blood Cell Oxidative Damage

In recent years, the focus of attention on curcumin in the hematological system has gradually expanded from “natural antioxidant” to “red blood cell storage protectant” and “delivery system optimization”. Hicks et al. used curcumin gel preparations to improve the oxidative damage of stored red blood cells, showing that curcumin delivery into red blood cells can reduce ferrylHb and restore ATP, which was important new evidence connecting curcumin with transfusion medicine [43]. Kang et al. used PVA to improve the solubility of curcumin and the free radical scavenging activity of AAPH, indicating that the carrier itself significantly affected the apparent activity of curcumin in the aqueous red blood cell model [44]. Yakubu et al., Jacob et al., and Bertoncini-Silva et al. summarized curcumin formulation strategies from the perspectives of nanodelivery, liposomes, nanoemulsions, polymer micelles, solid lipid nanoparticles, and nanostructured lipid carriers, providing a path for solving its water solubility and bioavailability problems [51,52,53].

## 5. Endpoint of Erythrocyte Experiments and Protective Effects of Curcumin

### 5.1. Protection of the Red Blood Cell Membrane System

The lipid peroxidation of the red blood cell membrane is a key damaging event in both the H_2_O_2_ and AAPH models. The peroxyl radicals(ROO∙) released by AAPH can seize hydrogen atoms from membrane lipids, forming lipid free radicals and initiating a chain reaction. H_2_O_2_ can also indirectly trigger lipid peroxidation through hydroxyl radicals(∙OH) and the oxidation products of hemoglobin via the pathway of hemoglobin. The final products of lipid peroxidation, MDA and 4-HNE, can further react with the amino and sulfhydryl groups of membrane proteins, altering the interactions of structural proteins such as band 3, spectrin, and protein 4.1 [54]. Banerjee et al. found in the AAPH model that curcumin could reduce lipid peroxidation and hemolysis of red blood cells, but its effect showed a bidirectional change with concentration, suggesting that curcumin could act as an antioxidant in the lipid membrane environment, or exhibit pro-oxidative characteristics under high concentration or specific oxidative conditions [40]. Zhang et al. used hemolysis rate, MDA and T-SOD as the main indexes in AAPH model of chicken red blood cells, and proved that curcumin could significantly reduce MDA production in the range of 0.5–20 μM in a clear dose-dependent manner [41,42]. Since chicken red blood cells are nucleated and contain mitochondria, this result belongs to the direct phenotypic evidence of avian red blood cells, and it cannot be inferred that mammalian mature red blood cells are induced by antioxidant enzyme transcription. This result indicates that curcumin, as a lipophilic polyphenol, can closely or integrate into the lipid bilayer of red blood cell membranes, exerting a chain-breaking antioxidant effect before peroxyl free radicals attack the membrane lipids. It is worth noting that the reduction in MDA cannot alone prove complete membrane recovery. It should also be combined with membrane fluidity, osmotic fragility, deformability, band 3 function, and electron microscopy/microscopic morphology results for interpretation.

The membrane fluidity and osmotic fragility of the red blood cell membrane reflect the combined state of membrane lipids and membrane scaffolds. Oxidative stress can cause membrane lipid saturation, an increase in lipid peroxidation products, and enhanced protein cross-linking, thereby leading to the hardening of the membrane, increased osmotic fragility, and an increased risk of hemolysis [29]. Maruyama et al. used an exogenous ROS generation system to cause acute oxidative damage and observed significant abnormalities in the rheological properties of human red blood cells, suggesting that oxidative damage directly altered the passage ability of red blood cells in the microcirculation [55]. In the H_2_O_2_ model, Snyder et al. proved that H_2_O_2_ exposure would lead to a decrease in red blood cell deformability, morphological changes, abnormal surface characteristics, and hemoglobin–membrane protein cross-linking [56]. The membrane protection of curcumin might come from hydrophobic interactions and the combined effect of anti-lipid peroxidation. Its aromatic ring and hydrophobic chain segments entered the hydrophobic region of the lipid bilayer, change the local microviscosity of the membrane, and made the membrane less sensitive to peroxyl free radical attack. Benincasa et al. observed in the H_2_O_2_ model that curcumin pretreatment significantly reduced hemolysis, indicating its ability to maintain membrane integrity in acute oxidative environments [38]. Hicks et al. in the blood storage injury model found that curcumin gel not only reduced hemoglobin oxidation but also restored ATP, suggesting that membrane stability and energy metabolism maintenance might mutually promote each other [43].

Red blood cell membrane protein oxidative damage includes band 3 aggregation, tyrosine phosphorylation, carbonylation, hemoglobin–membrane protein cross-linking, hemoglobin protein damage, and abnormal PRDX2 binding [27]. Remigante et al. detected morphology, oxidative stress parameters, and band 3-mediated SO_4_^2−^ transport in the AAPH-induced human red blood cell injury, finding that AAPH caused GSH depletion, lipid and protein oxidation, the formation of acanthocytes, abnormal distribution and hyperphosphorylation of band 3, and anthocyanin-enriched extract could partially protect the function of AE1 [36]. Although the research subject was not curcumin, it proved that band 3 was an important endpoint for membrane protein damage and natural antioxidant evaluation in the AAPH model. Hicks et al. further directly linked curcumin to the protection of band 3 through a curcumin gel study. This study treated and stored red blood cells with peroxide, and detected ferrylHb, ATP, and phosphorylation at the band 3 site. They found that the curcumin formulation could prevent the phosphorylation of band 3 at Y359 and Y21, and improve the oxidative indicators related to red blood cell storage damage [43]. This suggests that the pathway by which curcumin protects membrane proteins may include: reducing the high-valent oxidation state of hemoglobin, reducing the local ROS caused by membrane-bound hemoglobin, blocking the secondary modification of membrane proteins by membrane lipid peroxidation products, and maintaining the stability of ATP-dependent ion pumps and membrane scaffolds. As shown in Table 2, “direct evidence” means that the subject himself is treated with curcumin/oxidant and the endpoint of red blood cells is determined. Although chicken red blood cells are direct cell experiments, they are not classified as direct mechanism evidence of mature mammalian red blood cells because of their nuclei and mitochondria. The whole animal or clinical supplement is classified as indirect evidence in vivo.

### 5.2. Regulation of the Antioxidant System Within Red Blood Cells

The core function of the antioxidant enzyme system within red blood cells is to convert superoxide anion, H_2_O_2_, and organic peroxides into less toxic products. SOD catalyzes the disproportionation of O_2_∙^−^ to H_2_O_2_, while CAT and GSH-Px further eliminate H_2_O_2_, and PRDX2 plays an important role in the clearance of low-concentration peroxides. In the AAPH model, the continuous generation of free radicals gradually depletes GSH and inhibits the activity of antioxidant enzymes, leading to the accumulation of lipid peroxidation and membrane protein damage [23]. Zhang et al. found that curcumin pretreatment can maintain the T-SOD activity of chicken red blood cells after AAPH challenge [41]. This result is the direct cell phenotype of bird nucleated red blood cells, which can be explained as reducing the inactivation or consumption of existing enzymes, but it cannot be used to prove that *SOD* gene transcription or neosynthesis occurs in mature red blood cells of mammals. At the clinical and systematic evaluation level, Kavyani et al. conducted a systematic review and meta-analysis on the effects of curcumin supplementation on inflammatory, oxidative stress, and endothelial function indicators, and the results showed that curcumin improved multiple indicators such as SOD, GPx, CAT, MDA, CRP, IL-6, and TNF-α [57]. Hosseini et al. conducted a RCT meta-analysis of the combined use of curcumin and piperine, and found that combined supplementation increased SOD and GSH levels, and reduced MDA, TNF-α and IL-6 [58]. These studies are not direct red blood cell experiments, but they support the possibility that curcumin regulates the antioxidant defense in the overall redox environment.

GSH is one of the most important non-enzymatic antioxidants within red blood cells. Due to the lack of a nucleus and mitochondria in red blood cells, they rely on the pentose phosphate pathway to produce NADPH to maintain the reduced state of GSH. Therefore, the GSH/GSSG balance can reflect the red blood cell’s reducing capacity. Both AAPH and H_2_O_2_ can deplete GSH. When GSH decreases, the ability of GPx to clear peroxides weakens, protein sulfhydryl groups are prone to oxidation, and the stability of the membrane skeleton decreases [23]. Remigante et al. observed in the AAPH model that AAPH led to GSH depletion and accompanied by the decline in band 3 function and abnormal morphology [36]. The mechanisms by which curcumin protects GSH levels include directly reducing the free radical load, reducing the consumption of GSH for peroxide clearance, and possibly through the Nrf2/ARE regulation of GSH synthesis-related genes in nucleated cells. It must be emphasized that mature red blood cells cannot rely on the nuclear entry of Nrf2 to rapidly supplement GSH synthetase through new gene transcription, but the Nrf2 regulation in erythroid precursor cells, endothelial cells, liver, and immune cells can improve the systemic antioxidant environment of the body. Therefore, this article places the Nrf2/ARE evidence in an indirect mechanism, while the maintenance of GSH in mature red blood cells is mainly explained as “reducing consumption” and “protecting the existing enzyme system”.

The restoration of red blood cell redox homeostasis is not only manifested by the decrease in ROS, but also by the recovery of metabolic flux, membrane protein function, and energy status. Spinelli et al. pointed out that red blood cell oxidative stress can alter glycolysis, PPP, glutathione cycle, and purine metabolism. Once ATP and NADPH supply decreases, ion pumps, membrane skeleton and antioxidant enzymes will be affected [25]. Hicks et al. observed that curcumin gel treatment of peroxidized damaged red blood cells restored ATP, indicating that the protective effect of curcumin might go beyond simple free radical clearance and involve the coupling of energy metabolism and membrane stability [43].

### 5.3. Protection of Red Blood Cell Morphology and Function

After red blood cell oxidative damage, echinocytic, spherocytic, stomatocytic, wrinkled, vesiculated, and cell fragments often occur, AAPH-induced continuous lipid peroxidation can lead to the formation of scirrhous cells. H_2_O_2_-induced acute oxidation can cause membrane protein cross-linking and cell hardening. Remigante et al. reported in the AAPH model that the sickle-shaped red blood cell formation, abnormal distribution of band 3 and oxidative damage occurred simultaneously [36]. Martínez-Vieyra et al. conducted research on the red blood cells of patients with hypertension and found that oxidative stress was related to cytoskeleton reorganization and changes in membrane structure, suggesting that morphological changes in red blood cells were not only a phenomenon in an in vitro model but also reflected the microcirculation risk in chronic diseases [59].

The deformability of red blood cells is crucial for their passage through capillaries and maintenance of tissue oxygen supply. Hemoglobin oxidation, membrane lipid peroxidation, ATP decline, and cross-linking of membrane skeleton proteins all reduce deformability. Maruyama et al. demonstrated from the perspective of rheology that acute oxidative damage caused abnormal erythrocyte viscoelasticity and microfluidic transport ability [55]. Xu et al.’s review on the self-regulation of red blood cell oxygen transport pointed out that red blood cells regulated oxygen-carrying capacity through hemoglobin conformation, 2,3-BPG, metabolic allocation, and membrane mechanical state [60]. Therefore, if curcumin can simultaneously reduce hemoglobin oxidation, maintain ATP, and protect membrane structure, it may have indirect protection for oxygen transport efficiency.

Hemolysis is the ultimate manifestation after the loss of red blood cell membrane integrity and is the most common endpoint in H_2_O_2_ and AAPH models. AAPH-induced hemolysis usually has a time lag, reflecting the continuous accumulation of free radicals and the gradual reaching of the rupture threshold of membrane lipid peroxidation. The H_2_O_2_-induced hemolysis is more dependent on the concentration and the intensity of acute hemoglobin/membrane protein oxidation. The studies by Banerjee et al. and Zhang et al. jointly proved that curcumin reduced the AAPH-induced hemolysis [40,41,42]. The studies by Benincasa et al. and Hicks et al. supported that curcumin reduced hemolysis and improve storage quality in H_2_O_2_ or oxidatively damaged red blood cells [38,43]. However, the hemolysis rate itself cannot distinguish the upstream mechanisms such as membrane lipid peroxidation, hemoglobin oxidation, energy depletion, or ionic homeostasis imbalance.

## 6. Stratification of Mechanism Evidence: Direct Mechanism and Indirect Mechanism of Mammalian Mature Erythrocytes

### 6.1. Direct Antioxidant Mechanism

The free radical scavenging ability of curcumin originates from the hydrogen donation of phenolic hydroxyl groups and the stability of conjugated structures to free radicals [61]. Kang et al. found that curcumin significantly improved the scavenging ability of DPPH, ABTS, AAPH free radicals and NO after improving the water dispersion of curcumin using PVA. This indicates that water accessibility is an important variable determining the in vitro antioxidant reading of curcumin [44]. In the AAPH model of red blood cells, curcumin can directly capture ROO∙, delaying the propagation of lipid peroxidation chain. In the H_2_O_2_ model, it may reduce acute oxidative damage by eliminating ∙OH and other secondary free radicals generated by the Fenton reaction.

The β-diketone/eneol structure of curcumin enables it to have the ability to chelate metal ions such as Fe^2+^ and Cu^2+^ [62,63]. In red blood cells, iron not only exists in hemoglobin heme but may also participate in peroxidation reactions in a free or weakly bound form. Metal ion chelation can reduce the efficiency of H_2_O_2_ conversion to ∙OH, thereby reducing hemoglobin oxidation and membrane lipid peroxidation. The acute protective effect of curcumin shown in the H_2_O_2_ model can be partially explained by this mechanism, but the contribution size needs to be further verified through the iron chelator control, desferrioxamine positive control, and hemoglobin oxidation spectrum analysis [64].

Curcumin does not necessarily “eliminate all ROS.” More accurately, it can reduce oxidative pressure at the stages of ROS generation, diffusion, and chain amplification. In the H_2_O_2_ model, curcumin can reduce ferrylHb and membrane-bound hemoglobin-related ROS [65]. In the AAPH model, it reduces membrane internal free radical diffusion by capturing ROO∙ [66]. In the storage blood model, it reduces the oxidative energy metabolism imbalance by protecting ATP and band 3 [67]. The Nrf2 transcriptional regulation does not hold in mature red blood cells, but an indirect system of protection can form in nucleated cells and erythroid precursor cells.

### 6.2. Mechanism of Red Blood Cell Membrane Stability

**Figure 4:**
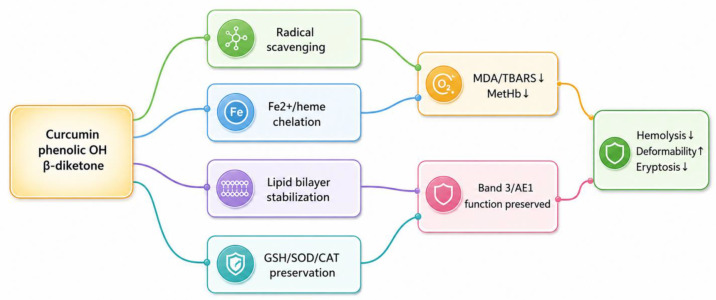
Curcumin protects erythrocytes through radical scavenging, Fe^2+^/heme chelation and Fenton reaction inhibition, membrane stabilization, and preservation of GSH, SOD, and CAT activity. These actions reduce MDA/TBARS and MetHb, maintain band 3 protein integrity, improve deformability, and ultimately suppress eryptosis and hemolysis.

Curcumin is hydrophobic and can easily be localized in the hydrophobic regions of the lipid bilayer. Its phenolic hydroxyl groups may be close to the membrane interface, thus being able to intercept lipid free radicals in the membrane phase and reduce the rate of membrane lipid peroxidation [68,69]. This localization explains why curcumin has a prominent protective effect on membrane lipid peroxidation and hemolysis in the AAPH model (Figure 4). It should be noted that the aggregation, precipitation, or carrier encapsulation of curcumin in the aqueous phase can significantly alter its efficiency in entering the membrane phase, so solvents and formulations are important variables in the design of red blood cell experiments [70].

Lipid peroxidation is a free radical chain reaction. Theoretically, one ROO∙ can trigger the continuous oxidation of multiple lipid molecules until it is terminated by an antioxidant. The chain-breaking effect of curcumin can be expressed as Cur-OH donating hydrogen to the lipid peroxide free radical, generating a more stable curcumin radical, thereby terminating the chain propagation [71]. The AAPH model provides the most intuitive verification environment: if curcumin significantly prolongs the half-time of hemolysis and reduces MDA, it suggests that it has successfully blocked the chain reaction in the membrane phase [72].

Band 3 and spectrin are the core structures for red blood cell membrane stability and deformability. Band 3 is both an anion exchange protein and a scaffold for the interaction of hemoglobin, glycolytic enzyme, and membrane skeleton. Hicks et al. demonstrated that curcumin gel prevented the phosphorylation of band 3 in stored red blood cells, and Remigante et al. demonstrated that AAPH reduced the activity of AE1/*SLC4A1* and caused the abnormal distribution of band 3 [36,43]. These studies collectively suggest that future research on the curcumin protection of red blood cells should not be limited to hemolysis and MDA, but should incorporate the function of band 3, tyrosine phosphorylation, membrane skeleton rearrangement, and red blood cell morphology as core tests.

### 6.3. Antioxidant Defense Within Red Blood Cells and Regulation of Energy Metabolism

The GSH/GSSG balance relies on the pentose phosphate pathway to provide NADPH. Early GSH depletion in the AAPH model can cause red blood cells to enter an “antioxidant defense deficit” state [73]. In the H_2_O_2_ model, if the H_2_O_2_ load exceeds the clearance capacity of CAT/GPx/PRDX2, GSH will be rapidly consumed. Curcumin can reduce free radical load and thereby decrease GSH consumption, maintaining a higher GSH/GSSG ratio [74]. This mechanism does not require transcriptional upregulation in red blood cells, making it more in line with the biology of mature red blood cells.

After reducing the free radical load, curcumin can protect the existing enzyme activity rather than inducing new enzyme expression. Zhang et al. detected T-SOD activity maintenance in the AAPH chicken red blood cell model, indicating that curcumin indirectly prevented SOD inactivation or excessive consumption after reducing oxidative attack [41]. This can be used as direct phenotypic evidence of birds’ red blood cells, but it can only support “preservation of existing enzyme activity” but not “gene expression induction” for mature mammalian red blood cells. In nucleated cells, curcumin can increase antioxidant enzyme expression through Nrf2/ARE.

ATP is crucial for maintaining the membrane skeleton, Na^+^/K^+^-ATPase, Ca^2+^-ATPase, membrane lipid asymmetry, and deformability in red blood cells. Oxidative stress can inhibit the key enzymes of glycolysis, leading to a decrease in ATP. The decrease in ATP will further aggravate ion imbalance, Ca^2+^ accumulation, cell shrinkage, and PS externalization. Hicks et al. observed that curcumin gel restored ATP in peroxidized damaged red blood cells [43], providing important evidence for the “curcumin–metabolic protection–membrane stability” mechanism.

### 6.4. Regulation of Eryptosis in Red Blood Cells

Eryptosis is a regulated form of death-like process in mature red blood cells under oxidative stress, hyperosmolarity, energy deficiency, or Ca^2+^ load, characterized by increased Ca^2+^, opening of Gardos channels, K^+^ efflux, water loss, and cell shrinkage. Tkachenko et al. emphasized that Ca^2+^ signaling is the core regulatory axis of eryptosis and suggested that at least detecting increased Ca^2+^ and PS externalization can reliably identify eryptosis [32]. Curcumin, by reducing ROS, protecting ATP and membrane stability, theoretically can indirectly inhibit Ca^2+^ influx and cell shrinkage, but further proof is needed through Annexin V, Fluo-4/Fluo-3, FSC and membrane vesicle detection.

PS externalization is an important signal for eryptosis and red blood cell clearance by macrophages and is also related to coagulation activation and particle release [75]. Oxidative stress can activate the Ca^2+^-dependent scramblase through Ca^2+^ dependence, weaken the flippase function due to ATP decline, and disrupt asymmetry of membrane lipids, promoting PS exposure [76]. If curcumin can maintain ATP, reduce ROS and protect membrane lipids, it may reduce PS externalization [77]. Future research should separately detect hemolysis and PS externalization, as hemolysis represents membrane rupture, while PS externalization represents that the red blood cell remains intact but has entered the clearance process.

Hemolysis and eryptosis can occur consecutively or compete with each other. Moderate oxidative damage may first induce PS externalization and red blood cell clearance. Strong oxidative damage can directly cause membrane rupture and hemolysis [78]. The reduction in hemolysis by curcumin does not necessarily mean inhibiting eryptosis, and the reduction in PS externalization does not necessarily mean preventing all membrane damage [77]. Therefore, in SCI research, it is recommended to simultaneously measure: free hemoglobin/hemolysis rate, Annexin V positive rate, cell volume, Ca^2+^, MDA, GSH, ATP, and membrane protein oxidation [79].

### 6.5. Evidence of Indirect Mechanism In Vivo and Nucleated Cells: Nrf2/ARE, Inflammation and Other Cell Models

The Nrf2/ARE pathway is an important transcriptional regulatory system for antioxidant responses in nucleated cells. Curcumin can affect the cysteine status of Keap1, inhibit Nrf2 degradation, promote Nrf2 nuclear translocation, and upregulate the expression of antioxidant genes such as *HO-1*, *NQO1*, *GCLC*, *SOD*, *CAT*, and *GPx*. Qi et al. found in the H_2_O_2_-induced HTR8/SVneo trophoblast cell model that a low dose of curcumin can reduce ROS, enhance antioxidant enzyme activity, and activate Nrf2, and the protective effect weakened after knockdown of Nrf2 [80]. Guo et al. also reported in corneal endothelial cells that curcumin can resist oxidative damage through the Keap1/Nrf2/ARE pathway [81]. These pieces of evidence can be regarded as indirect support for the antioxidant potential of the curcumin system, but cannot directly claim that Nrf2 nuclear translocation occurs in mature red blood cells. If there are Nrf2/HO-1, mitochondria or apoptosis-related changes in nucleated red blood cells of birds such as chickens, they can only be used as direct cellular evidence of bird red blood cells and cannot be extrapolated to the mechanism of mature red blood cells in mammals. Therefore, this pathway is only used here to explain the antioxidant regulation of systemic or progenitor cells, and should not be used as a direct transcription mechanism in mature red blood cells.

The inflammatory microenvironment can increase the oxidative burden of red blood cells through neutrophil oxidative burst, myeloperoxidase, free hemoglobin, complement, and cytokines. Curcumin in clinical meta-analyses can reduce inflammatory indicators such as CRP, IL-6, and TNF-α [57,58] and inhibit NF-κB and inflammationosome-related pathways in various cell models [50]. For red blood cells, these mechanisms are more likely to provide the indirect protection by reducing external oxidative/inflammatory stress, rather than occurring a complete inflammatory signal transduction within the red blood cells. As shown in Table 3, it presents the evidence for the direct and indirect mechanisms by which curcumin protects red blood cells.

Non-hematopoietic cell models provide rich information on the antioxidant mechanism of curcumin, including Nrf2/ARE, NF-κB, SIRT1, SIRT3-SOD2, mitochondrial autophagy, and caspase. However, mature red blood cells have no cell nucleus and mitochondria, so these mechanisms can only be used to explain the indirect protection in erythroid precursor cells, endothelial cells, immune cells, or tissue microenvironments. Therefore, “directly” in this review always means that the experimental object is mature mammalian red blood cells and the endpoint is measured in the cell or on the membrane. Neither “in vivo” nor “nucleated cells” evidence is used to prove transcription, mitochondria or classical apoptosis pathways in mature red blood cells.

## 7. Evidence Integration and Comparison of H_2_O_2_ and AAPH Models

### 7.1. Experimental Research Evidence in H_2_O_2_-Induced Model

The core feature of the H_2_O_2_ model is hemoglobin oxidation and acute membrane damage. Benincasa et al. established an accelerated aging model by treating human red blood cells with H_2_O_2_ and detected the hemolysis rate and TBARS. The conclusion was that the curcumin pretreatment could completely or significantly prevent the H_2_O_2_-induced hemolysis and performed outstandingly in maintaining the integrity of the red blood cell membrane [38]. Hicks et al. further detected ferrylHb in peroxidation damage and stored red blood cells, proving that the curcumin gel could reduce pro-oxidative high hemoglobin and restore ATP [43]. In both studies, curcumin/preparation was given directly to isolated or stored human mature red blood cells and the endpoint of red blood cells was determined. Therefore, it belongs to the direct evidence of mature red blood cells in mammals. However, its conclusion is limited to in vitro or storage conditions and cannot be automatically equated with in vivo clinical effects.

H_2_O_2_ can simultaneously induce hemoglobin oxidation and membrane lipid peroxidation. Remigante et al.’s research on quercetin, although not curcumin, provided a standardized detection framework for H_2_O_2_ human red blood cell models: ROS, MDA, GSH, antioxidant enzyme, morphology, and membrane fragility were used to evaluate the antioxidant effect of natural polyphenols [37]. Hicks’ curcumin research incorporated band 3 phosphorylation into the evaluation of storage injury, showing that curcumin could prevent the abnormal phosphorylation of the key tyrosine site of band 3 [43]. Therefore, the protection of curcumin against H_2_O_2_ damage should be understood as a comprehensive effect of “hemoglobin oxidation inhibition + membrane protein protection + energy metabolism maintenance”.

Currently, studies directly evaluating the effect of curcumin on the deformability of human red blood cells induced by H_2_O_2_ are still limited, but related models indicate that H_2_O_2_ damage can significantly affect the morphology and mechanical properties of red blood cells [30,82]. In the future, microfluidics, ektacytometry, optical tweezers, or atomic force microscopy should be combined with traditional biochemical indicators to clarify whether curcumin can improve the ability of red blood cells to pass through capillaries at the rheological level. This is particularly crucial for the application of curcumin in blood storage and transfusion medicine [38].

### 7.2. Experimental Research Evidence in AAPH-Induced Model

The typical evidence of curcumin in the AAPH model mainly comes from the research of Banerjee and Zhang [40,41,42]. The in vitro experiment of human mature red blood cells by Banerjee et al. is direct evidence, showing that low to medium concentration reduces hemolysis and lipid peroxidation, while high concentration may promote oxidation. The experiment of chicken red blood cells by Zhang et al. belongs to the direct cellular evidence of bird nucleated red blood cells, which can support the improvement of chicken red blood cell membrane damage. The results of broiler supplementation belong to indirect evidence in vivo and cannot be used to prove the transcription or mitochondrial mechanism in mature red blood cells of mammals.

In the AAPH model, an increase in MDA, a decrease in GSH, and a decrease in SOD activity usually precede massive hemolysis. Zhang et al. found that curcumin could reduce MDA and restore T-SOD [41]. Remigante et al. demonstrated in the AAPH human red blood cell model that AAPH could lead to GSH depletion, lipid peroxidation, and band 3 function decline [36]. These studies suggest that the protection of curcumin against AAPH damage should be elaborated as “early GSH protection–MDA reduction–band 3/membrane morphology maintenance–hemolysis delay” as a logical chain. The conclusions of AAPH that can be directly supported in mature red blood cells of mammals should be limited to the measured endpoints such as hemolysis, lipid peroxidation, GSH, ion release and band 3. T-SOD or apoptosis index of chicken red blood cells should be listed as non-equivalent levels.

Compared with other natural antioxidants, the advantage of curcumin lies in its strong lipid solubility, which makes it easy to be localized in the membrane phase. Its disadvantage is poor water solubility and stability. Zheng et al. studied the protection of mulberry leaf flavonoids against AAPH-induced oxidative damage in sheep red blood cells and found that flavonoids could inhibit hemolysis and lipid peroxidation [83]. Wang et al. studied the morphological protection effect of a new peptide in AAPH-induced oxidative damage in human red blood cells, suggesting that the AAPH red blood cell model has been widely used to evaluate different antioxidants [84]. Compared with BDMC, curcumin showed a stronger erythrocyte protective effect in the study conducted by Zhang et al., indicating that the methoxy, phenolic hydroxyl, and β-diketone structures jointly affect the antioxidant efficiency [42].

### 7.3. Commonalities and Differences in the Protective Effects of Curcumin in the Two Models

The common effects of curcumin in the two models include free radical scavenging, MDA reduction, maintenance of membrane integrity, and decrease in hemolysis rate. Regardless of whether the oxidant is H_2_O_2_ or AAPH, it ultimately causes membrane lipid peroxidation and membrane protein damage, so the protection of curcumin on the membrane phase is its common mechanism. The doses of curcumin, carriers, and pretreatment times in different studies vary, resulting in differences in the results. Future experiments should report the experimental conditions in μM concentration, hematocrit, oxidant concentration, and treatment time.

The H_2_O_2_ model is more suitable for discussing iron chelation, hemoglobin oxidation, ferrylHb and band 3 phosphorylation. The AAPH model is more suitable for discussing ROO∙ scavenging, membrane lipid peroxidation and half-life of hemolysis. If researchers only use one of the models, the conclusion should be limited to the corresponding damage type. When the goal is to prove that curcumin has broad-spectrum protection against red blood cell oxidative damage, two models should be combined and multiple indicator evidence chains should be used.

Model selection affects the judgment of curcumin effects. In the AAPH model, the insufficient water solubility of curcumin may lead to an underestimated effective concentration. In the H_2_O_2_ model, an excessively high oxidant concentration may mask the protective effect of the antioxidant. Additionally, the solvent DMSO and ethanol are capable of altering cell membrane fluidity. Furthermore, PVA, liposomes, micelles and gels can simultaneously change the accessibility and membrane localization of curcumin. The PVA study by Kang et al. and the gel study by Hicks et al. illustrate that the delivery system is no longer just a technical detail, but a key variable in the study of curcumin’s red blood cell protection [43,44].

## 8. Limitations and Future Prospects

### 8.1. Main Limitations of Current Research

The current evidence regarding curcumin’s protection against H_2_O_2_ and AAPH-induced oxidative damage in red blood cells mainly comes from in vitro models. In vitro models can precisely control the concentration of oxidants and the duration of treatment, but they cannot fully simulate the factors such as plasma protein binding, hepatic and intestinal metabolism, oxidative burst in immune cells, spleen clearance, and microcirculation shear force in the body. Clinical meta-analysis suggested that curcumin could improve MDA, SOD, GPx, CAT and inflammatory markers [85,86,87]. These results belong to systemic indirect evidence in vivo and cannot be directly equated with the red blood cell protection. Future studies need to validate red blood cell-specific indicators in samples from healthy volunteers, anemia, diabetes, chronic inflammation, stored blood, and blood transfusion patients. Therefore, the existing data support the rationality and preclinical potential of the mechanism, but it cannot prove the established clinical efficacy of curcumin in protecting red blood cells in human body.

Low water solubility, low absorption, rapid metabolism, and limited tissue distribution of curcumin are the main bottlenecks for its translational application [88,89]. Bertoncini-Silva et al. pointed out that multiple nanocarriers significantly increased the relative bioavailability of curcumin, but the pharmacokinetic differences between different carriers were significant [53]. For the red blood cell research, the bioavailability issue is also manifested as the difficulty of uniform dispersion of curcumin in vitro experiments, resulting in an uncertain real contact concentration [90].

Curcumin has a bidirectional effect in red blood cell models, with high concentrations possibly promoting oxidation or disturbing membrane structure [91,92]. Therefore, future research should clearly define the low, medium, and high concentration ranges and evaluate safety using cytotoxicity, permeability fragility, hemolysis, PS externalization, and membrane fluidity as indicators [38,93]. Clinically, the interaction of curcumin with piperine, anticoagulants, antiplatelet drugs, or liver enzyme substrates also needs to be considered [94,95].

Currently, there is a lack of research on curcumin’s red blood cell protection using clinical blood samples. For example, the oxidative stress characteristics of red blood cells in patients with diabetes, thalassemia, sickle cell disease, G6PD deficiency, and chronic kidney disease are different [96,97,98,99], and the protective effect of curcumin may have significant individual differences. Future research should compare the sensitivity of red blood cells from different diseases to H_2_O_2_ and AAPH [100,101] and detect whether curcumin can improve the deformability, PS externalization, MDA, GSH, MetHb, and band 3 function of patient-derived red blood cells [102,103,104].

### 8.2. Research Progress of New Delivery Systems

Nanocarriers are the main strategy for improving water solubility, bioavailability, and cellular accessibility of curcumin [105]. Yakubu et al. summarized that the systems such as liposomes, nanoemulsions, polymer nanoparticles, and solid lipid nanoparticles enhanced the solubility and targeted delivery of curcumin [51]. Jacob et al. summarized that nanocarriers improved the stability, cellular uptake, and therapeutic effect of curcumin [52]. Alshammari et al. emphasized that biocompatible nanocarriers had potential in functional foods and biomedical applications [106]. For red blood cell models, the carrier not only determines whether curcumin enters the membrane phase but may also affect the membrane itself, so a carrier blank control should be set. PVA, liposomes, hydrogels, micelles and nanoparticles can independently change erythrocyte membrane fluidity, osmotic fragility, PS externalization, hemolysis and colorimetric/fluorescence readings. Before attributing the protective effect to curcumin, each preparation study should include carrier blank, solvent control, curcumin-free carrier control, dose-dependent blood compatibility test, and washing/residue evaluation if necessary.

Curcumin derivatives and metabolites may have higher stability and different membrane affinity [107,108]. Pandey et al. studied the reducing form of curcumin metabolites and pointed out that tetrahydrocurcumin and others might have stronger antioxidant stability [47]. Zhang et al. found through comparing curcumin and BDMC that structural differences affected the antioxidant effect of red blood cells [42]. In the future, the roles of curcumin, DMC, BDMC, tetrahydrocurcumin, and metal complexes in the H_2_O_2_/AAPH red blood cell model can be compared.

Complementary antioxidant formulations can utilize antioxidant agents in the aqueous phase and the membrane phase. Benincasa et al. compared curcumin with vitamin C and vitamin E combinations, suggesting that different natural compounds produced differential protection in the H_2_O_2_ red blood cell model [38]. Piperine increased the exposure of the curcumin system, and Hosseini et al. showed through a Meta-analysis that the combination of curcumin and piperine improved indicators such as SOD, GSH, and MDA [58,64]. In the red blood cell model, the combined formula should simultaneously evaluate the synergistic protection and potential pro-oxidative risks [109], as shown in Table 4.

### 8.3. Future Research Directions

In the future, proteinomics, metabolomics, lipomics and erythrocyte membrane protein phosphorylationomics should be utilized to systematically analyze the protective effects of curcumin [110]. D’Alessandro et al. demonstrated that the red blood cell omics study indicated that red blood cell storage damage involved metabolic, lipid, and protein modification networks [111]. The research on curcumin can adopt isotope tracing analysis of PPP fluxes, LC-MS detection of GSH/GSSG and lipid peroxides [112], and phosphorylated protein group detection of band 3 and membrane skeleton protein changes [30].

During the red blood cell storage, ATP levels decrease, 2,3-BPG is depleted, oxidative damage occurs, particulates are released, membranes become harder, and hemolysis increases [113,114,115]. Anastasiadi et al. pointed out that enhancing antioxidant capacity is an important strategy for improving the quality of stored red blood cells [116]. Tran et al. compared different storage solutions and emphasized the balance between metabolic support, pH buffering, prevention of oxidative damage, and osmotic regulation [117]. Hicks et al. studied the curcumin gel as one of the candidate protective agents for stored red blood cells [43]. At present, the storage application based on curcumin should be described as a candidate strategy, which requires standardized blood bag unit research, residue test, blood compatibility evaluation and blood transfusion safety evaluation.

Red blood cells are not isolated. Red blood cell oxidative damage can release free hemoglobin, heme, ATP, particulates, and DAMPs, further affecting endothelium, platelets, leukocytes, and coagulation [118,119,120]. Lam et al. proposed that red blood cells participated in innate immunity activation through TLR9 binding to DNA [121]. Ren et al. believed that red blood cells were an unignored member of the immune system [122]. Weisel and Litvinov emphasized that red blood cells play an important role in hemostasis and thrombosis [123]. Therefore, the translational significance of curcumin red blood cell protection lies not only in reducing hemolysis, but also in reducing inflammation and the thrombosis microenvironment. However, until the direct data of patient-derived red blood cells and clinical outcomes are obtained, the systematic significance still belongs to the hypothesis generation stage.

There are significant differences in red blood cell oxidative stress and antioxidant reserves among different populations. Older adults, diabetes, chronic inflammation, G6PD deficiency, thalassemia, and long-term stored red blood cells may have different responses [124,125,126,127,128,129]. Yadav et al. studied the potential of red blood cells as biomarkers of aging, suggesting that red blood cell indices, deformability and oxidative stress markers reflected the state of human aging [130]. Future curcumin intervention should combine baseline oxidative stress levels, pharmacokinetics, red blood cell membrane characteristics, and disease types to avoid the simplistic strategy of “one dose fits all”.

### 8.4. Conclusions

In summary, H_2_O_2_ and AAPH provide complementary but distinct models of RBC oxidative damage: H_2_O_2_ mainly reflects acute hemoglobin/iron-dependent injury, whereas AAPH mainly reflects sustained peroxyl-radical-driven membrane lipid peroxidation. Curcumin may protect RBCs through free radical scavenging, metal ion chelation, membrane stabilization, preservation of antioxidant defenses, protection of hemoglobin/band 3, ATP maintenance, and hemolysis reduction. However, direct evidence in mature mammalian RBCs remains limited and should be distinguished from evidence from avian nucleated RBCs, animal supplementation, clinical supplementation, or non-RBC cell models. Future studies should use standardized H_2_O_2_/AAPH conditions, physiologically relevant curcumin doses, appropriate carrier controls, and RBC-specific endpoints before translating these findings to blood storage, transfusion medicine, or precision nutrition. The proposed future research directions are summarized in Table 5.

Multi-dimensional endpoint and omics technology were used to evaluate curcumin delivery system in strict control, safety testing and hypothesis-driven ways in blood storage, transfusion medicine and precision nutrition. It cannot be regarded as a verified clinical application.

## Figures and Tables

**Table 1:** Comparison of oxidative damage models of red blood cells induced by H_2_O_2_ and AAPH.

Comparison of Projects	H_2_O_2_ Model	AAPH Model	Significance of Evaluation for Curcumin
Free radical	When H_2_O_2_ enters red blood cells, it undergoes a reaction involving Fe^2+^ and Hb to produce ∙OH; this reaction is extremely rapid and intense.	The thermal decomposition of AAPH generates alkyl radicals which combine with oxygen to form ROO∙, releasing a relatively continuous amount of energy.	Evaluation of Fe^2+^ chelation, Hb oxidation suppression, and acute membrane protection (H_2_O_2_), and chain-breaking anti-lipid-peroxidation activity (AAPH).
Main injury target	Hemoglobin, methemoglobin, ferrylHb, membrane protein, band 3 and cytoskeleton.	Membrane lipids, polyunsaturated fatty acids, GSH, band 3, ion pumps and membrane morphology.	Support for curcumin’s radical-scavenging, membrane-stabilizing, and antioxidant-preserving effects.
Common indicators	MetHb, ferrylHb, TBARS/MDA, hemolysis rate, ATP, band 3 phosphorylation, morphology.	Hemolysis time curve, half-time of hemolysis, MDA, GSH, SOD, CAT, AE1/SO4^2−^ transport.	Combined assessment of hemolysis, MDA, GSH/SOD/CAT, MetHb, band 3, and PS externalization is recommended over single-absorbance readouts.
Methodology	Optimize H_2_O_2_ concentration and exposure time to control acute toxicity and prevent nonspecific lysis.	Correct for AAPH-induced Hb spectral interference and ensure proper dispersion of lipophilic samples.	Curcumin has poor water solubility. DMSO, ethanol, PVA, liposome or gel carriers may all affect the interpretation of the results.

**Table 2:** Hierarchical summary of evidence in core experimental research of curcumin and related preparations.

RBC Model	Modeling Oxidant; Curcumin Concentration/Preparation Type	Endpoints	Conclusions	Reference
Human red blood cells	AAPH; free curcumin concentration gradient (<10 μM can protect GSH; Lipid peroxidation and hemolysis IC_50_ are about 23.2 and 43 μM)	Hemolysis, lipid peroxidation, K^+^ release, GSH.	Low-to-moderate doses were protective; high doses may be pro-oxidant.	Banerjee et al., 2008 [40]
Chicken red blood cells	AAPH; curcumin 0.5–10 μM.	Hemolysis rate, MDA, T-SOD	Curcumin can reduce hemolysis and MDA, increase T-SOD, and have time and dose-dependent properties.	Zhang et al., 2014 [41]
Chicken red blood cells and broiler chicken model	AAPH 75 mM; 0.5 μM, 1 μM, 5 μM, 10 μM and 20 μM of curcumin in chicken erythrocytes; Broiler diet 150 mg/kg	Hemolysis, MDA, SOD and intracellular antioxidant indicators.	Curcumin is superior to BDMC overall, especially in maintaining SOD and reducing MDA	Zhang et al., 2019 [42]
Human red blood cells	H_2_O_2_; 100 μM curcumin; compared with vitamin C/E and combined treatment	Hemolysis rate, TBARS.	100 μM curcumin can significantly prevent H_2_O_2_-induced hemolysis.	Benincasa et al., 2025 [38]
Human red blood cells	Peroxide/storage; 100 μM curcumin gel preparation	FerrylHb, ATP, band 3 phosphorylation	Curcumin reduces ferrylHb, restores ATP, and prevents band 3 phosphorylation.	Hicks et al., 2024 [43]
No RBC model	Red blood cell oxidation model has not been established; PVA improves dispersion and stability of curcumin.	Solubility, stability, AAPH free radicals, NO, ABTS/DPPH.	PVA enhances the stability of curcumin and the free radical clearance activity of AAPH.	Kang et al., 2024 [44]

**Table 3:** Evidence attributes of curcumin-mediated erythrocyte protection.

Mechanism Category	Evidence Attribute in Mature Red Blood Cells of Mammals	Representative Evidence/Methods
Free radical scavenging, inhibition of membrane lipid peroxidation.	Direct experimental evidence. No need for nucleus or mitochondria.	AAPH/H_2_O_2_ red blood cell model; measurement of hemolysis, MDA, TBARS, GSH.
Chelation of iron ions and inhibition of hemoglobin oxidation.	Direct experimental evidence. Related to Hb, Fe^2+^, ferrylHb and MetHb.	H_2_O_2_ model; detection of MetHb, ferrylHb, Hb oxidation spectrum.
Band 3, spectrin, membrane skeleton protection.	Direct experimental evidence. Direct evidence of red blood cell membrane structure.	AE1/SO_4_^2−^ transport, phosphorylation, protein carbonylation, immunoblotting.
Nrf2/ARE, HO-1 expression upregulation.	Can only be used as nucleated cells or indirect evidence in vivo.	Nucleated cells, erythroid precursor cells, tissue models and clinical systemic indicators.
Mitochondrial membrane potential, classical caspase apoptosis.	Cannot be established. Bird red blood cells or other nucleated cells should be stratified separately.	Nucleated cell model; mature red blood cells retain only some caspase-related proteins.
Activation of inflammatory corpuscles, inflammatory cytokines and NF-κB reaction	Indirect mechanism. RBCs are influenced by inflammatory microenvironments but do not execute nucleated-cell transcriptional inflammatory programs.	Nucleated immune/endothelial/tissue model and inflammatory markers of clinical system.
Changes in Nrf2/HO-1, mitochondria or apoptosis in bird nucleated erythrocytes	Direct cellular evidence of avian erythrocytes. Non-equivalent level or indirect support for mature red blood cells of mammals.	Chicken erythrocytes and broilers. Separately from the endpoint of human/mammalian mature red blood cells.

**Table 4:** The combined research direction of curcumin delivery system and red blood cell oxidative damage study.

Delivery Strategy	Main Advantages	Potential Problems in Red Blood Cell Models	Suggested Tests
PVA dispersion system.	Improve water solubility, suspension stability and AAPH free radical scavenge activity.	PVA may change the contact mode of curcumin with the membrane.	PVA blank, solvent control, particle size/dispersibility, hemolysis, MDA, GSH.
Liposome/phospholipid complex.	Promote membrane phase delivery and is suitable for fat-soluble curcumin.	The liposome itself can fuse with erythrocyte membrane or change membrane fluidity.	Liposome blank, membrane fluidity, osmotic fragility, carrier blank control.
Polymeric micelle/nanoparticles.	Improve stability and controlled release.	The compatibility between nanomaterial and red blood cells need to be verified.	Nanoparticle blank, hemolysis rate, PS externalization, particle size, and Zeta potential.
Gel preparation.	Suitable for sustained-release delivery under blood storage conditions.	Clinical conversion requires assessment of wash, residue, and infusion safety.	Gel blank, washing/residue test, phosphorylation of ferrylHb, ATP and band 3, and storage hemolysis.
Compound antioxidant formula.	Simultaneously covers aqueous-phase ROS and membrane-phase peroxyl radicals.	There may be dose interactions and oxidation promotion windows.	Single component blank, carrier blank, synergistic index, dose response, MDA, MetHb, PS eversion.

**Table 5:** Proposed research directions for the future.

Research Direction	Recommended Design	Key Indicators	Expected Value
Standardized in vitro model.	Parallel H_2_O_2_/AAPH models using same-source RBCs; standardize hematocrit, oxidant dose, and exposure time.	Hemolysis, MDA, GSH, SOD/CAT/GSH-Px, MetHb, ATP, band 3, PS externalization.	Improved comparability across studies and clarification of the effective dose range of curcumin.
Erythrocyte mechanics and microcirculation function.	Combining microfluidic, ektacytometry and micromorphology.	Deformation index, transit time, cell morphology, osmotic fragility.	From biochemical protection to functional protection.
Formulation delivery studies.	Free curcumin, PVA, liposome, micelle, gel and derivative were compared.	Water dispersibility, membrane localization, cell compatibility, hemolysis, and PS externalization.	Solve problems of wat solubility and stability of curcumin.
Blood storage conversion.	A safe delivery system was added under standard blood storage conditions and a dynamic follow-up of 42 days was performed.	ATP, 2,3-BPG, ferrylHb, band 3, microparticles, hemolysis rate.	Preclinical support for transfusion and blood preservation; safety, residue, and hemocompatibility validation required.
Clinical sample validation.	Comparison of health, diabetes, anemia, G6PD deficiency, aged samples.	Baseline oxidative stress, curcumin response, pharmacokinetics, and safety.	Establish a precise antioxidant intervention basis.

## Data Availability

No new data were created or analyzed in this study. Data sharing is not applicable.

## Abbreviations

The following abbreviations are used in this manuscript:

RBCs	Red blood cells
ROS	Reactive oxygen species
RNS	Reactive nitrogen species
H_2_O_2_	Hydrogen peroxide
AAPH	2,2′-azobis(2-methylpropionamidine) dihydrochloride
Nrf2	Nuclear factor erythroid 2-related factor 2
ARE	Antioxidant response element
HO-1	Heme oxygenase-1
GSH	Glutathione
SOD	Superoxide dismutase
ATP	Adenosine triphosphate
MDA	Malondialdehyde
CAT	Catalase
GSH-Px	Glutathione peroxidase
PRDX2	PRDX2
PS	Phosphatidylserine
GPx	Glutathione peroxidase
TBARS	Thiobarbituric acid reactive substances
GSSG	Oxidized glutathione
T-SOD	Total superoxide dismutase
BDMC	Bisdemethoxycurcumin
PVA	Polyvinyl alcohol
DPPH	2,2-diphenyl-1-picrylhydrazyl
ABTS	2,2′-azino-bis(3-ethylbenzothiazoline-6-sulfonic acid)
NO	Nitric oxide
·OH	Hydroxyl radical
ROO·	Peroxyl radical
MetHb	Methemoglobin
AE1	Anion exchanger 1
DMSO	Dimethyl sulfoxide
NF-κB	Nuclear factor kappa B
MAPK	Mitogen-activated protein kinase
NOX	NADPH oxidase
CRP	C-reactive protein
IL-6	Interleukin-6
TNF-α	Tumor necrosis factor-alpha
RCT	Randomized controlled trial
NADPH	Reduced nicotinamide adenine dinucleotide phosphate
PPP	Pentose phosphate pathway
2,3-BPG	2,3-bisphosphoglycerate
*SLC4A1*	Solute carrier family 4 member 1
Na^+^/K^+^-ATPase	Sodium–potassium adenosine triphosphatase
Ca^2+^-ATPase	Calcium adenosine triphosphatase
Fluo-4/Fluo-3	Fluo-4/Fluo-3 calcium fluorescent indicators
FSC	Forward scatter
*NQO1*	NAD(P)H: quinone oxidoreductase 1
*GCLC*	Glutamate–cysteine ligase catalytic subunit
HTR8/SVneo	HTR8/SVneo trophoblast cell line
SIRT1	Sirtuin 1
SIRT3-SOD2	Sirtuin 3–superoxide dismutase 2
G6PD	Glucose-6-phosphate dehydrogenase
DMC	Demethoxycurcumin
LC-MS	Liquid chromatography–mass spectrometry
DAMPs	Damage-associated molecular patterns
TLR9	Toll-like receptor 9
DNA	Deoxyribonucleic acid
